# The [4Fe4S] Cluster of Yeast DNA Polymerase ε Is Redox Active and Can Undergo DNA-Mediated Signaling

**DOI:** 10.1021/jacs.1c07150

**Published:** 2021-09-24

**Authors:** Miguel N. Pinto, Josy ter Beek, Levi A. Ekanger, Erik Johansson, Jacqueline K. Barton

**Affiliations:** † Division of Chemistry and Chemical Engineering, California Institute of Technology, Pasadena, California 91125, United States; ‡ Department of Medical Biochemistry and Biophysics, Umeå University, SE-910 87 Umeå, Sweden; § Department of Chemistry, The College of New Jersey, Ewing, New Jersey 08628, United States

## Abstract

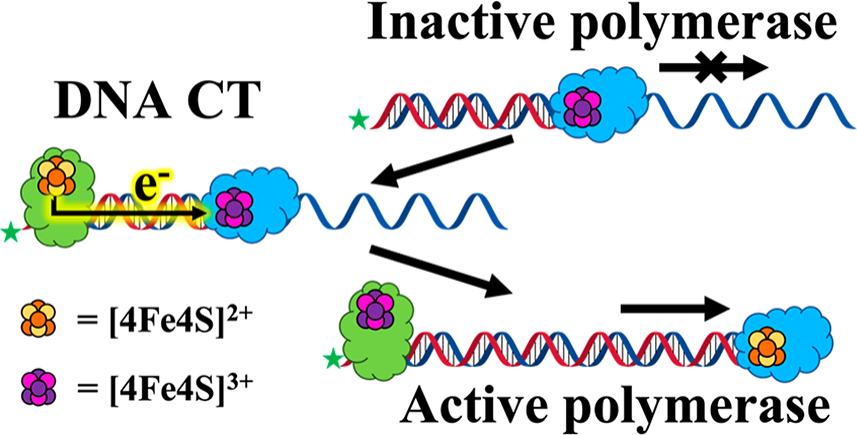

Many DNA replication and DNA repair enzymes have been found to carry [4Fe4S] clusters. The major leading strand polymerase, DNA polymerase ε (Pol ε) from *Saccharomyces cerevisiae*, was recently reported to have a [4Fe4S] cluster located within the catalytic domain of the largest subunit, Pol2. Here the redox characteristics of the [4Fe4S] cluster in the context of that domain, Pol2_CORE_, are explored using DNA electrochemistry, and the effects of oxidation and rereduction on polymerase activity are examined. The exonuclease deficient variant D290A/E292A, Pol2_CORE_exo^–^, was used to limit DNA degradation. While no redox signal is apparent for Pol2_CORE_exo^–^ on DNA-modified electrodes, a large cathodic signal centered at −140 mV vs NHE is observed after bulk oxidation. A double cysteine to serine mutant (C665S/C668S) of Pol2_CORE_exo^–^, which lacks the [4Fe4S] cluster, shows no similar redox signal upon oxidation. Significantly, protein oxidation yields a sharp decrease in polymerization, while rereduction restores activity almost to the level of untreated enzyme. Moreover, the addition of reduced EndoIII, a bacterial DNA repair enzyme containing [4Fe4S]^2+^, to oxidized Pol2_CORE_exo^–^ bound to its DNA substrate also significantly restores polymerase activity. In contrast, parallel experiments with EndoIII^Y82A^, a variant of EndoIII, defective in DNA charge transport (CT), does not show restoration of activity of Pol2_CORE_exo^–^. We propose a model in which EndoIII bound to the DNA duplex may shuttle electrons through DNA to the DNA-bound oxidized Pol2_CORE_exo^–^ via DNA CT and that this DNA CT signaling offers a means to modulate the redox state and replication by Pol ε.

## Introduction

All organisms require genome replication with a high degree of fidelity and have evolved complex molecular machinery to accomplish the task.^1^ Eukaryotic DNA replication is carried out by B-family polymerases, and it is initiated by DNA primase and DNA polymerase (Pol) α, followed by elongation of the leading and lagging strands by Pol ε and Pol δ, respectively.^2^ Pol ε and Pol δ are highly processive multisubunit enzymes composed of a catalytic subunit and regulatory subunits.^2,3^ The catalytic domain of Pol ε and Pol δ exhibits both polymerase and 3′-5′ exonuclease (proofreading) activities.^2−5^

Pol ε is the largest of the replicative polymerases in *Saccharomyces cerevisiae*, and it is composed of four subunits, Pol2 (256 kDa), Dpb2 (79 kDa), Dpb3 (23 kDa), and Dpb4 (22 kDa).^5^ The catalytic subunit, Pol2, is a flexible two-lobed structure that contains the N-terminal domain (NTD) in lobe 1 and the C-terminal domain (CDT) in lobe 2. The NTD catalyzes DNA polymerization and proofreading nuclease activity, whereas the CTD is noncatalytic. The intrinsically high processivity of Pol ε arises in part from two insertions (∼100 residues each) in the NTD of Pol2 that envelop the nascent DNA double strand.^5,6^ Pol ε processivity is also modestly increased through interactions with proliferating cellular nuclear antigen (PCNA) and the two accessory subunits, Dpb3 and Dpb4.^3,5^ In contrast, the smaller enzyme Pol δ (3 subunits, 220 kDa total) does not have a domain that encircles the nascent DNA strand and only becomes highly processive when bound to the PCNA replication clamp.^2,6^ The functions of the three accessory subunits in Pol ε include mediating interactions between the polymerase and the DNA duplex and/or other biological molecules.^5−7^

An important feature of eukaryotic DNA replication and repair enzymes, including polymerases, is the presence of highly conserved cubane [4Fe4S] clusters.^5,8−11^ Iron–sulfur clusters are common redox cofactors found in or near the active sites of enzymes in all forms of life.^11^ These metal cofactors appear to be ubiquitous in DNA and RNA processing enzymes,^10^ with the most recent example being the RNA-dependent RNA polymerase of the severe acute respiratory syndrome coronavirus 2 (SARS-CoV-2).^10a^ Eukaryotic Pol ε and Pol δ also contain a [4Fe4S] cluster within their catalytic subunit.^4,5^ Initial literature reports suggested that the role of the [4Fe4S] cluster of DNA processing enzymes is primarily structural in nature. However, structural requirements can be addressed using alternative methods, such as simple Zn^2+^ cations, that would obviate the need for a metabolically expensive [4Fe4S] cofactor.^8^ Traditionally, the roles of [4Fe4S] clusters in biology have focused on electron transfer.^8−11^ As with other cluster-containing DNA processing enzymes, the [4Fe4S] of Pol ε does not directly catalyze redox transformations during its enzymatic activity.^4,5^ Recent studies have shown instead that redox-active [4Fe4S] cofactors in DNA processing enzymes offer a means to modulate DNA binding and therefore have strong implications in DNA replication.^8,12^

The redox potentials of [4Fe4S] clusters in DNA repair enzymes shift to a physiologically relevant range (∼90 mV vs NHE) when bound to the DNA polyanion; it is this shift in potential that leads to a redox switch for binding DNA.^8^ In addition, electrons can migrate rapidly through duplexed DNA, allowing for long-range charge transport (CT).^13^ DNA-mediated redox signaling has been found between DNA-bound enzymes with [4Fe4S] cluster oxidation states of 2+ and 3+.^8^ Studies have demonstrated that DNA repair proteins may take advantage of DNA CT to scan the genome efficiently and identify lesions, mismatches, or other perturbations. Redox-active [4Fe4S] clusters are also utilized for substrate handoff in yeast and human primase through DNA CT.^12^ For yeast Pol δ, the redox-active [4Fe4S] cluster provides a means to modulate polymerase activity reversibly; DNA CT from guanine radicals, generated under conditions of oxidative stress, can lead to oxidation of the [4Fe4S] cluster in Pol δ, inhibiting replication, but rereduction of the cluster restores replication activity.^12^ While DNA CT appears to play an important role in the activity of various polymerases, no studies on DNA synthesis by Pol ε have been reported within the context of DNA CT to/from the enzyme-bound [4Fe4S] cluster.

Here we report on the redox chemistry of the [4Fe4S] cluster in yeast Pol ε bound to DNA and its effects on polymerase activity. We examined the DNA electrochemistry of Pol2_CORE_exo^–^, an exonuclease-deficient truncation of the catalytically active subunit of Pol ε.^5a^ Pol2_CORE_ is a polymerase- and exonuclease-active truncation of Pol2 (residues 1–1228) corresponding to the NTD. In addition, Pol2_CORE_ is known to contain the [4Fe4S] cluster within a cysteine-rich domain “CysX”, composed of residues C665, C668, C667, and C763.^5a^ We examined the exonuclease deficient (exo^–^) D290, E292A mutant of Pol2_CORE_, to limit DNA degradation by the polymerase.^5a^

## Results and Discussion

### DNA Electrochemistry

**Figure 1:**
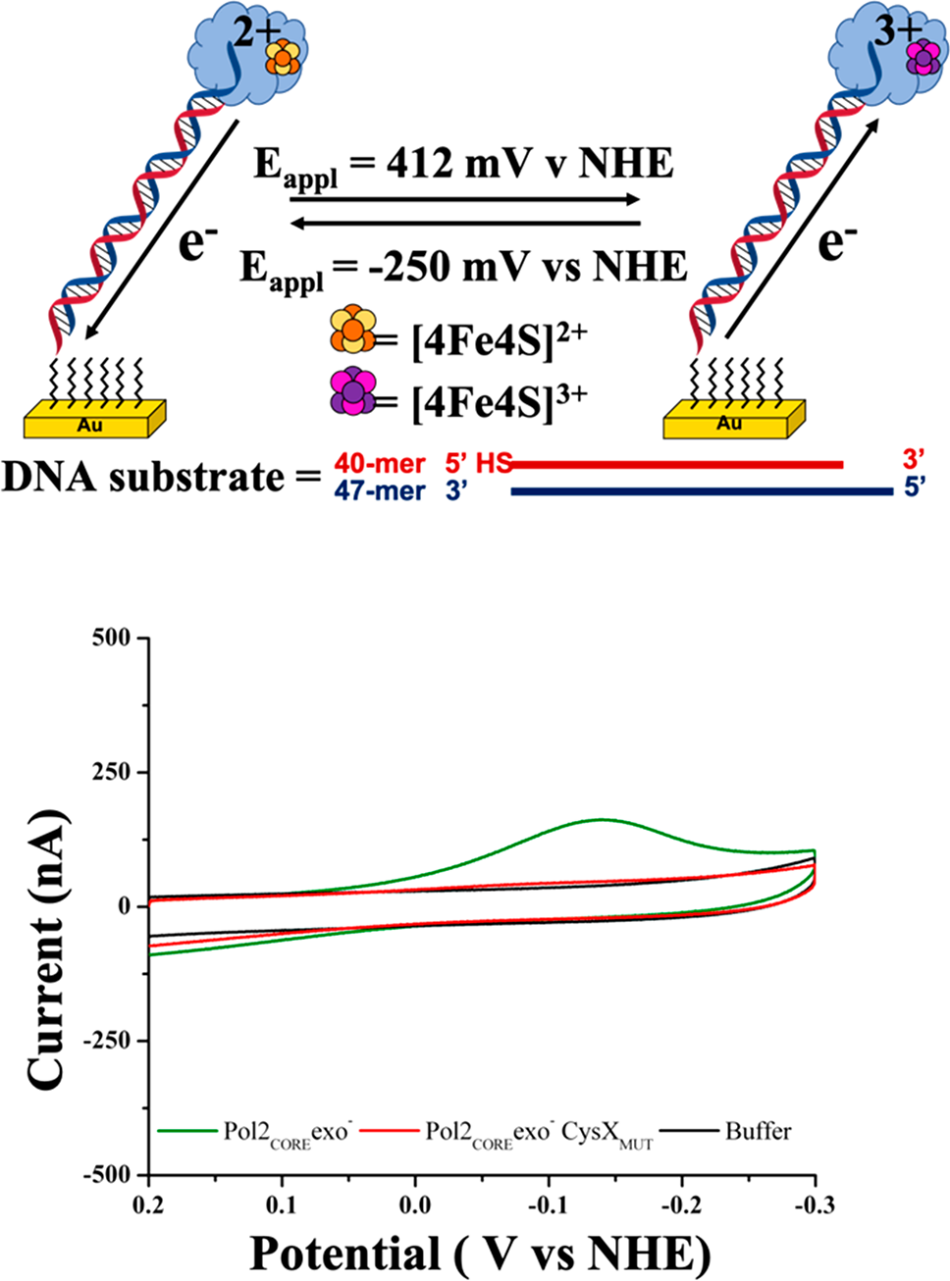
(Top panel) Electrochemical oxidation of Pol2_CORE_exo^–^ using DNA-modified electrodes. DNA substrate is attached to gold (Au) surface through a 5′ alkanethiol group. Complementary matched DNA strand is slightly longer, yielding a 7-nt overhang that serves as the natural binding substrate for Pol2_CORE_exo^–^. (Bottom panel) CV scans of electrochemically oxidized Pol2_CORE_exo^–^ (5 μM) exhibit a large cathodic CV signal centered around −140 V vs NHE (green trace), but no signal is observed when Pol2_CORE_exo^–^CysX_MUT_ (red trace) or buffer (5 mM NaH_2_PO_4_, 50 mM NaCl, pH 7.0; black trace) is electrochemically oxidized. Potential applied (*E*_appl_) for bulk oxidation is 412 mV vs NHE for 500 s. CV scan rate = 100 mV s^–1^. Pol2_CORE_exo^–^ protein sample concentrations used for cyclic voltammetry experiments are 5 μM (ε[4Fe4S]_410_ = 17 000 M^–1^ cm^–1^).

Cyclic voltammetry (CV) experiments were performed using DNA-modified gold electrodes to characterize the DNA-bound redox potentials of Pol2_CORE_exo^–^ (Figure 1 and Figures S1–S3). DNA-modified electrodes are an exceptional tool for the clean one-electron oxidation and/or reduction of [4Fe4S]-containing enzymes bound to DNA using DNA CT. By use of this system, electrons can be shuttled from the Au electrodes through the duplexed DNA, oxidizing or reducing the [4Fe4S] cluster of Pol2_CORE_exo^–^ depending on the applied potential (*E*_appl_; Figure 1 top panel).

CV scans of electrochemically unaltered Pol2_CORE_exo^–^ (5 μM) samples did not show detectable cathodic or anodic signals on the DNA-modified electrodes (Figure S1). This result contrasts with CV scans on yeast Pol δ bound tightly with PCNA, where the cluster is redox-active on the DNA-modified electrode and exhibits a reversible electrochemical signal with a midpoint potential of 113 ± 5 mV vs NHE.^12a^ It was originally expected that a similar reversible electrochemical signal might be observed for Pol2_CORE_exo^–^, since Pol ε bears similarities to Pol δ, and Pol2_CORE_exo^–^ retains strong DNA-binding affinity.^12a^

Bulk electrolysis experiments were then performed to generate oxidized Pol2_CORE_exo^–^ (Figure 1 and Figure S1). A large cathodic signal centered at −140 mV vs NHE was observed for Pol2_CORE_exo^–^ after bulk oxidation (500 s, *E*_appl_ = 412 mV vs NHE; Figure 1 bottom panel, green trace). These findings resemble those obtained using human and yeast DNA primase, where electrochemically unaltered protein exhibits no signal, but the application of positive potentials (412 or 512 mV vs NHE) produces a large cathodic signal centered at −140 mV vs NHE.^12b,12c^

Our DNA electrochemical studies included a double cysteine to serine mutant (C665S/C668S; CysX_MUT_) of Pol2_CORE_exo^–^ (Figure 1 and Figure S2). As mentioned above, the [4Fe4S] cluster of Pol2 is in a cysteine rich domain called CysX within Pol2_CORE_. Studies have shown that Pol2_CORE_exo^–^CysX_MUT_ does not bind a [4Fe4S] cluster and its polymerase activity is severely compromised.^5a,5d^ Haploid yeast cells expressing Pol ε CysX_MUT_ have been demonstrated to be inviable.^5a^ CV scans of Pol2_CORE_exo^–^CysX_MUT_ using DNA-modified electrodes do not show a significant cathodic signal, even after bulk oxidation (500 s, *E*_appl_ = 412 mV vs NHE; Figure 1 bottom panel, red trace). The absence of redox activity of Pol2_CORE_exo^–^CysX_MUT_ can thus be explained by the absence of the [4Fe4S] cluster. Bulk reduction (500 s, *E*_appl_ = −250 mV vs NHE) of protein and buffer samples also did not yield significant cathodic or anodic signals (data not shown).

### Effects of Redox State on Polymerase Activity

**Figure 2:**
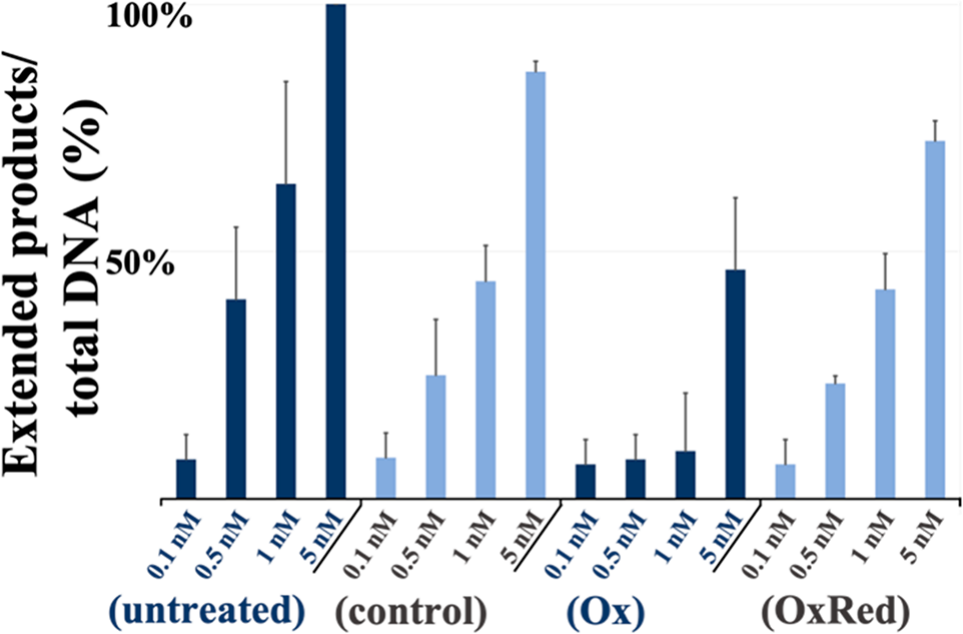
Bar graph summarizing averaged denaturing PAGE results of Pol2_CORE_exo^–^ primer extension assay upon [4Fe4S] cluster oxidation/reduction. (untreated) activity of untreated Pol2_CORE_exo^–^: (control) activity of Pol2_CORE_exo^–^ after incubation on DNA modified electrodes without applied potential; (Ox) activity of Pol2_CORE_exo^–^ after bulk oxidation (600 s, *E*_appl_ = 412 mV vs NHE); (OxRed) activity of Pol2_CORE_exo^–^ after bulk oxidation (600 s, *E*_appl_ = 412 mV vs NHE) followed by bulk rereduction (600 s, *E*_appl_ = −250 mV vs NHE). The quantification of DNA products (as % of total) was obtained by dividing the signal of the total amount of extended products by the total amount of primer used (sum of all extended product signals and unextended primer signal). All experiments were carried out in triplicate; error bars indicate standard deviation.

These CV results prompted us to investigate the effect of oxidation of the [4Fe4S] cluster in Pol2_CORE_exo^–^ on its polymerase activity. A primer extension assay was utilized^5a^ where a prewarmed solution containing dNTPs was mixed with a Pol2_CORE_exo^–^ solution containing a DNA template with a primer that could be elongated. The DNA substrate was composed of a 5′ fluorescein-labeled DNA primer (20-mer) annealed to a complementary template strand (50-mer). The conditions of the primer-extension assay were optimized to determine the concentration range (0.1–5 nM) of Pol2_CORE_exo^–^ that produced detectable, consistent, and reliable results. Primer extension products were separated using 20% denaturing urea polyacrylamide gel electrophoresis, imaged using a Typhoon scanner, and analyzed using ImageQuant software. These results are summarized in Figures 2, S4, and S5.

Electrochemically untreated Pol2_CORE_exo^–^ yielded complete extension of the fluorescein-labeled DNA primer at a concentration of 5 nM. Pol2_CORE_exo^–^ samples with oxidized [4Fe4S] clusters (Ox) were prepared by performing bulk oxidation (600 s, *E*_appl_ = 412 mV vs NHE) on the DNA-modified electrodes. Protein concentrations (based on [4Fe4S]) had to be optimized (20 μL, 20 nM) since samples containing high concentrations of Pol2_CORE_exo^–^ (over 50 nM) or high-volume samples (over 25 μL) contained enough electrochemically unaltered protein (after bulk oxidation) to produce a strong product signal. In addition, we employed DNA-modified gold electrodes with large surface areas (*A* = 0.16 cm^2^), rather than our multiplexed chips, to decrease the amount of electrochemically unaltered protein remaining in solution. We estimate the yield of oxidized protein by taking the difference between the total charge obtained in the presence of Pol2_CORE_exo^–^ and the charge generated by electrolysis of the buffer alone giving >95% on average (Figure S4; see figure caption for calculation of bulk oxidation yield). Bulk electrolysis experiments were carried out in the absence of oxygen to prevent aerobic degradation of the clusters.^12a^ Oxidized Pol2_CORE_exo^–^ samples showed a clear and dramatic decrease in replication when compared to Pol2_CORE_exo^–^ treated similarly on the electrodes but without oxidation (control; Figure 2 and Figure S5).

We then investigated whether a rereduction of the previously oxidized Pol2_CORE_exo^–^ samples would also influence polymerase activity. Samples of oxidized Pol2_CORE_exo^–^ were prepared as described above and then treated under bulk reduction conditions (600 s, *E*_appl_ = −250 mV vs NHE) on the same electrode (OxRed Pol2_CORE_exo^–^). Primer extension assays of rereduced (OxRed) Pol2_CORE_exo^–^ revealed significant restoration of the polymerase activity (Figure 2). It should be noted that primer extension assays of Pol2_CORE_exo^–^ samples, subjected to incubation on the DNA-modified electrodes for equivalent time but without an applied potential (control), consistently show decreased levels of polymerization compared to untreated Pol2_CORE_exo^–^ (Figure 2 and Figure S5). Likely, the observed discrepancy is due to protein loss during the procedure on the electrode because of protein remaining bound to the DNA-modified electrode, as well as protein mechanically lost during removal from the electrode. These results suggest that oxidation of the [4Fe4S] of Pol2_CORE_exo^–^ results in the observed reversible inhibition of replication, resembling results previously obtained using Pol δ.^12a^ Thus, the reversible oxidation and reduction of [4Fe4S] clusters in polymerases might provide a route through which polymerase activity is regulated.

It may be useful to consider how this inhibition of replication with oxidation may occur. As with other DNA-processing proteins containing [4Fe4S] clusters, the cluster is located far from the catalytic site in Pol2, and it is difficult to understand how cluster oxidation could affect catalysis. However, with other DNA-processing proteins, cluster oxidation was seen to increase binding to the DNA substrate significantly (>100×); in the case of highly processive polymerases, such tight binding could inhibit replication.

### DNA CT Signaling between DNA-Bound Proteins

Next we asked whether the redox state of Pol2_CORE_exo^–^ and, as a result, its DNA polymerase activity could be changed through DNA CT with a DNA-binding repair enzyme containing a reduced [4Fe4S]^2+^ cluster. We employed endonuclease III (EndoIII), an established DNA CT-proficient base excision repair glycosylase from *Escherichia coli*.^8,14^ Could these two DNA-binding enzymes from different organisms function chemically as redox signaling partners to modulate DNA synthesis?

**Figure 3:**
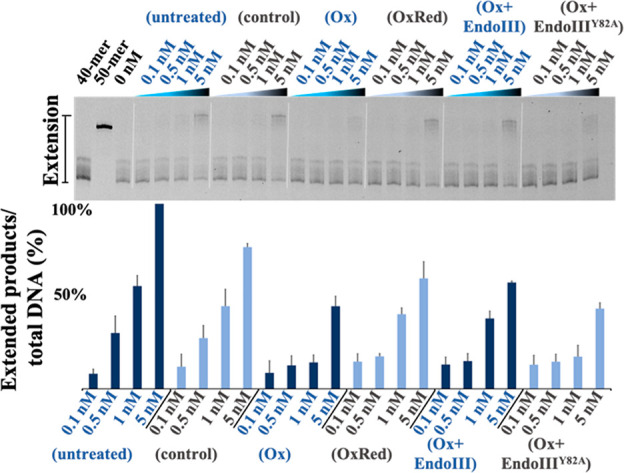
Pol2_CORE_exo^–^ primer extension assay results upon electrochemical oxidation/reduction using DNA-modified electrodes, and effect of DNA-mediated CT on the activity of oxidized Pol2_CORE_exo. (Top panel) Representative example of quantitative Pol2_CORE_exo^–^ primer extension assay results on denaturing PAGE. (Bottom panel) Bar graph summarizing averaged results of Pol2_CORE_exo^–^ primer extension assay: (untreated) activity of untreated Pol2_CORE_exo^–^; (control) activity of Pol2_CORE_exo^–^ after incubation with DNA modified electrodes without an applied potential; (Ox) activity of Pol2_CORE_exo^–^ after bulk oxidation (600 s, *E*_appl_ = 412 mV vs NHE); (OxRed) activity of Pol2_CORE_exo^–^ after bulk oxidation (600 s, *E*_appl_ = 412 mV vs NHE) followed by bulk rereduction (600 s, *E*_appl_ = −250 mV vs NHE). (Ox+EndoIII) is the activity of oxidized Pol2_CORE_exo^–^ after incubation with 50 nM EndoIII. (Ox+EndoIII^Y82A^) is the activity of oxidized Pol2_CORE_exo^–^ after incubation with 50 nM EndoIII^Y82A^. It should be noted that experiments with Ox, Ox+EndoIII, and Ox+EndoIII^Y82A^ were performed using the same oxidized Pol2_CORE_exo^–^ sample. Bulk oxidation and bulk reduction were performed on 20 μL of 20 nM Pol2_CORE_exo^–^ using DNA-modified electrodes. All experiments were carried out in triplicate; error bars indicate standard deviation.

To accommodate Pol2_CORE_exo^–^ and EndoIII binding onto the same dsDNA, we increased the length of the primer/template DNA substrate. The footprint of each protein is ∼10 base pairs. The DNA substrate was composed of a 5′ fluorescein-tagged 40-mer primer (instead of the original 20-mer) which was annealed to a 50-mer complementary DNA strand, resulting in 40 nucleotides of double-stranded DNA. An identical set of experiments as the ones described for Figure 2 were carried out for Pol2_CORE_exo^–^ (untreated, control, Ox, and OxRed) using the longer primer:template DNA adduct (Figure 3). Ox and OxRed samples of Pol2_CORE_exo^–^ were prepared using DNA-modified electrodes before carrying out the primer extension assays. Results (untreated, control, Ox, and OxRed) using the 40:50 primer:template DNA substrate (Figure 3) follow similar patterns as extension assays using the 20:50 primer:template substrate. As expected, upon varying the primer length, we again observe a decrease in replication activity for the oxidized Pol2_CORE_exo^–^(Ox) compared to both control and untreated samples. Similarly, we see that rereduction of oxidized Pol2_CORE_exo^–^ on the DNA electrode (OxRed) restores most of the activity.

We then asked whether EndoIII could serve as the reductant bound to DNA. The oxidized Pol2_CORE_exo^–^ sample prepared (20 μL, 20 nM) was diluted with buffer (5 mM NaH_2_PO_4_, 50 mM NaCl, pH 7.0) to appropriate concentrations, combined with the DNA substrate, and then mixed with either oxidized Pol2_CORE_exo^–^ sample (Ox) or oxidized Pol2_CORE_exo^–^ sample and EndoIII (Ox+EndoIII) to reach the final concentrations (0.1–5 nM Pol2_CORE_exo^–^); the solution was then incubated at ambient temperature before mixing with the prewarmed dNTPs to initiate the polymerase assay. The concentrations of EndoIII used were fixed at 50 nM (based on the concentration of [4Fe4S] cluster), 1 order of magnitude higher than the highest concentration of Pol2_CORE_exo^–^ used. The results (Figure 3) indicate that incubation of oxidized Pol2_CORE_exo^–^ with EndoIII restores polymerization to similar levels as those obtained when the protein is reduced on DNA-modified electrodes (compare Ox+EndoIII with OxRed, Figure 3). Still higher concentrations of EndoIII (250 nM) gave similar results (Figure S6). Overall, the activity of Ox+EndoIII is consistently slightly lower in activity than OxRed but higher than the oxidized sample. Again, we attribute these variations to the difficulty in removing oxidized protein from the electrode. As a control, we then included the variant EndoIII^Y82A^, which has similar enzymatic activity and binds to DNA with the same affinity as wt EndoIII but is deficient in carrying out DNA CT.^8,12e,14d^^e^ EndoIII^Y82A^ was used following the same protocols as EndoIII (Ox+EndoIII^Y82A^). The results with the CT-deficient mutant show little if any increase in activity relative to oxidized Pol2_CORE_exo^–^. These results taken together thus support the ability of the EndoIII protein to interact with Pol ε through DNA CT, yielding a restoration of polymerase activity upon cluster reduction to the 2+ form.

**Figure 4:**
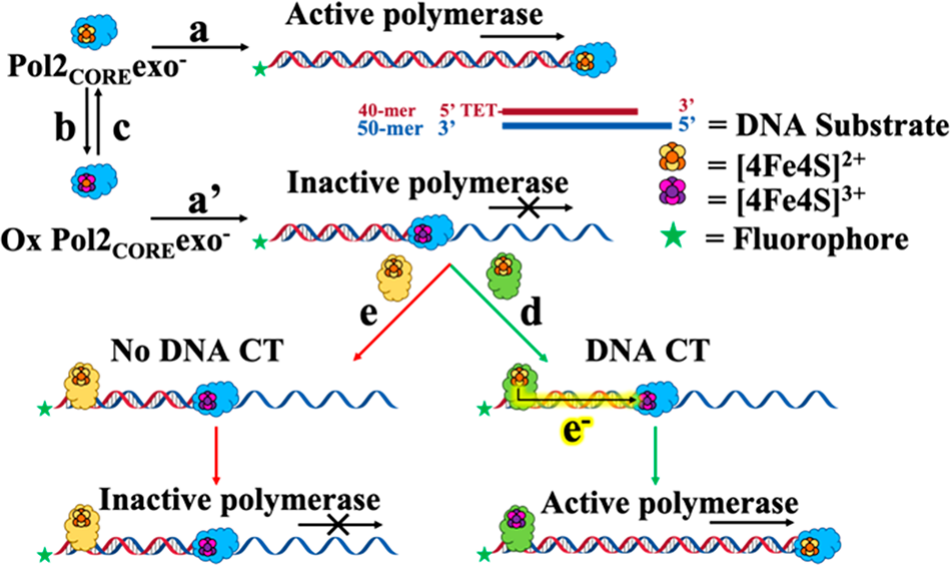
Model for DNA-mediated redox signaling between DNA processing enzymes Pol2_CORE_exo^–^ and EndoIII. (a) Pol2_CORE_exo^–^ in the presence of a DNA substrate and dNTPs has normal polymerase activity. (b) Bulk oxidation using DNA-modified electrodes produces oxidized Pol2_CORE_exo^–^ (a′). Oxidized Pol2_CORE_exo^–^ shows diminished polymerization in the presence of DNA substrate and dNTPs. (c) Rereduction of oxidized Pol2_CORE_exo^–^ by bulk electrolysis using DNA-modified electrodes restores Pol2_CORE_exo^–^ polymerization activity. (d) EndoIII is CT proficient and can reduce oxidized Pol2_CORE_exo^–^ through DNA-mediated redox signaling. (e) EndoIII^Y82A^ is CT-deficient and cannot reduce DNA-bound and oxidized Pol2_CORE_exo^–^; therefore the oxidized Pol2_CORE_exo^–^ remains inactive.

A model is presented in Figure 4, where Pol2_CORE_exo^–^, the exonuclease deficient and truncated catalytic subunit of Pol ε, shows normal polymerase activity when presented with dNTPs and a DNA-primer extension substrate (Figure 4a). Bulk oxidation of Pol2_CORE_exo^–^ using DNA-modified electrodes results in a decrease in polymerase activity with fewer replication products (b → a′, Figure 4). Bulk reduction of the oxidized Pol2_CORE_exo^–^ sample using DNA-modified electrodes can restore replication (b → c → a, Figure 4). But also addition of excess EndoIII similarly restores the polymerase activity through DNA-mediated redox signaling (b → a′ → d, Figure 4). In contrast, addition of EndoIII^Y82A^, deficient in DNA CT, shows no restoration of polymerase activity; here DNA CT between proteins is not available and Pol2_CORE_exo^–^ remains inactive (b → a′ → e, Figure 4). Redox control of Pol ε would thus provide interesting opportunities and is intriguing to consider, but the *in vivo* mechanism and possible partners still require further investigation.

## Conclusion

Taken together, the results reported here illustrate that the [4Fe4S] cluster of Pol2_CORE_exo^–^ is redox-active when the protein is bound to DNA and that the cluster oxidation state affects the polymerase activity reversibly; oxidation of the cluster inhibits DNA synthesis. Significantly, DNA-mediated CT may occur between a repair protein, also containing a redox-active [4Fe4S], and Pol2_CORE_exo^–^ to restore the polymerase activity of Pol ε. Redox signaling through DNA CT thus has the potential to rapidly modulate replication by Pol ε.

## Experimental Section

### General Methods

All reagents were obtained from commercial sources and used as received unless stated otherwise. Water used to prepare buffer solutions was purified on a Milli-Q Reference Ultrapure Water Purification System. Electrochemistry experiments used a standard three-electrode cell composed of multiplexed Au chip or a continuous Au(111) surface (0.16 cm^2^) bearing DNA modification as the working electrode, an Ag/AgCl reference electrode in 3 M NaCl (BASInc.), and a 1 mm diameter Pt wire (Lesker) as the counter electrode.^12,13^ Potentials were converted from Ag/AgCl to NHE by adding 212 mV to the potentials measured by the Ag/AgCl; this conversion accounted for both ambient temperature and the use of 3 M NaCl for reference storage.^12a^ To prevent cluster degradation in the presence of O_2_, all electrochemical manipulations and polymerase activity assays involving oxidized or reduced Pol2_CORE_exo^–^ samples were carried out under strict anaerobic conditions in vinyl chambers (glove bags) kept at atmospheres of 2–4% H_2_ in argon or N_2_ (≤1 ppm of O_2_) with Pd scrubbing towers (Coy Laboratories). Buffers were degassed by bubbling argon for a minimum of 2 h and stored under anaerobic conditions. UV–vis data were acquired using a Cary 100 Bio (Agilent) spectrophotometer. SDS–PAGE gel images were acquired using a Typhoon FLA 9000 (GE), and the resulting images were analyzed using ImageQuant software.

### Protein Purifications

1xFLAG-tagged Pol2_CORE_exo^–^ and Pol2_CORE_exo^–^ CysX_MUT_ were expressed in yeast and initial purification via M2 resin as previously described for full-length Pol ε.^5a^ . 1 mM DTT (instead of TCEP) was added to the elution fractions, which were then concentrated on a 50 kDa cutoff filter (Amicon) and loaded onto a Superose 12 PC 3.2/30 column (GEHealthcare) equilibrated with 25 mM HEPES, pH 7.6, 10% glycerol, 300 mM NaAc, 1 mM DTT, and 0.005% NP-40. *E. coli* EndoIII and EndoIII^Y82A^ were expressed and purified according to previously published protocols.^15a^

### General DNA Preparation

All oligonucleotides were purchased from Integrated DNA Technologies (IDT) and purified by reverse-phase high performance liquid chromatography using a C-18 column (Agilent). Masses of purified oligonucleotides were confirmed by MALDI-TOF/TOF mass spectrometry (Bruker AutoFlex) using MALDIAnalyzer ionization. Oligonucleotides containing thiol modifications were obtained from IDT in their disulfide form and were chemically reduced using 50-fold excess of tris(2-carboxyethyl)phosphine HCl and purified using Micro BioSpin columns pre-equilibrated with DNA buffer (50 mM NaCl, 5 mM NaH_2_PO_4_, pH 7.0) prior to annealing. DNA strands were annealed (1:1, 50 μM oligonucleotides in 100 μL) for 5 min at 90 °C followed by cooling to ambient temperature over 90 min in argon-sparged DNA buffer (50 mM NaCl, 5 mM NaH_2_PO_4_, pH 7.0). Annealed DNA samples were kept at −20 °C, used within 1 week, and thawed immediately before use.

### DNA Electrochemistry

DNA-modified electrodes^12−15^ were prepared by deprotecting and purifying a 40-mer ssDNA bearing a 5′-thiol modification and annealing it to a complementary well-matched 47-mer ssDNA. Annealing of these two strands yields a 7-nucleotide overhang that may serve as a natural binding site for Pol2_CORE_exo^–^. The resulting double stranded DNA (dsDNA) substrate was incubated overnight on a set of multiplexed gold electrodes to produce self-assembled low-density DNA monolayers, with surface coverage of about 15–20 pmol cm^–2^ with respect to dsDNA (determined by established protocols^16^). The electrode surfaces were then washed, passivated using β-mercaptohexanol, and rinsed again using degassed DNA buffer (5 mM NaH_2_PO_4_, 50 mM NaCl, pH 7.0). The electrode subassembly was brought into the anaerobic chamber and connected to the potentiostat equipped with a multiplexer (CH Instruments). Pol2_CORE_exo^–^ samples, originally stored at −80 °C, were then brought into the anaerobic chamber and thawed immediately before the start of electrochemical characterizations. The concentrations of Pol2_CORE_exo^–^ samples used for electrochemical characterizations were first adjusted to 5 μM based on [4Fe4S] cluster absorbance (ε[4Fe4S]_410_ = 17 000 M^–1^ cm^–1^). Cyclic voltammetry (CV; 100 mV/s scan rate) and square wave voltammetry (SQWV; 15 Hz frequency, 25 mV amplitude) scans were performed on 25 μL samples of buffer (5 mM NaH_2_PO_4_, 50 mM NaCl, pH 7.0), Pol2_CORE_exo^–^, or Pol2_CORE_exo^–^ CysX_MUT_ each using one separate quadrant of the multiplexed electrode subassembly.

### DNA Substrates for Electrochemistry

40-mer with 5′ thiol modification: 5′ HS-GTG CTG CAA CGT GTC TGC GCG CTG AGT GCA CGC AAC TCG C 3′.

47-mer (well matched): 5′ CTG TCG TGC GAG TTG CGT GCA CTC AGC GCG CAG ACA CGT TGC AGC AC 3′.

### Primer Extension Assays

Immediately prior to activity assays, a sample of Pol2_CORE_exo^–^ (20 nM) was prepared and aliquoted (20 μL each). One aliquot was kept without manipulation (untreated), one was incubated in the DNA-modified electrodes without an applied potential (control), another one was electrochemically oxidized (Ox; 600 s, *E*_appl_ = 412 mV vs NHE), and a fourth was electrochemically oxidized and reduced (OxRed; 600 s, *E*_appl_ = 412 mV vs NHE followed by 600 s, *E*_appl_ = −250 mV vs NHE) using a continuous DNA-modified Au(111) surface electrode (*A* = 0.16 cm^2^) electrodes. Low sample volumes (20 μL) and continuous Au-electrode wafers were employed to increase the yield of bulk oxidized/reduced Pol2_CORE_exo^–^ and therefore decrease the amount of electrochemically unaltered protein. The time that Pol2_CORE_exo^–^ samples were incubated on DNA-modified electrodes was kept constant (1 h) across all samples for every experiment.

The enzymatic activities of Pol2_CORE_exo^–^ (untreated, control, Ox, and OxRed) were investigated using a primer extension assay adapted from an established protocol.^5a^ Briefly, reaction mixture A (Mix A), containing untreated, control, Ox, or OxRed Pol2_CORE_exo^–^, DNA substrate (20 nM; 20:50 primer:template), Tris-HCl (20 mM, pH 7.8), sodium acetate (40 mM), and bovine serum albumin (BSA, 0.1 mg mL^–1^), was prepared and kept on ice. Reaction mixture B (Mix B), containing Tris-HCl (20 mM, pH 7.8), magnesium acetate (16 mM), BSA (0.1 mg mL^–1^), and dNTP mix (0.2 mM each), was prepared, aliquoted, and preincubated for at least 20 min at 30 °C. The primer extension assay was initiated by addition of 10 μL of Mix A to 10 μL of prewarmed Mix B followed by incubation at 30 °C for 10 min. The activity assay was terminated by addition of 20 μL of stop solution (95% formamide, 20 mM EDTA, 0.1% bromophenol blue, 0.05% xylene cyanol). Primer extension products were separated on a 20% denaturing urea polyacrylamide gel at 90 W for 2.5 h and visualized by fluorescence imaging using a Typhoon FLA 9000.

Primer extension assays using EndoIII and EndoIII^Y82A^ as the redox signaling partner were carried out as described above with the following modifications. Reaction mixture A′ (Mix A′) employed the use of a 40:50 primer:template DNA substrate (instead of 20:50) and contained EndoIII or EndoIII^Y82A^ (50 nM). All other concentrations, volumes, and conditions were kept constant. Visual discrimination between starting primer and extended product required an increase in SDS–PAGE separation time from 2.5 to 3.5 h. It is important to note that activity assays Ox, Ox+EndoIII, and Ox+EndoIII^Y82A^ were performed using the same electrochemically oxidized (600 s, *E*_appl_ = 412 mV vs NHE) Pol2_CORE_exo^–^ sample. Primer extension assay investigating two different concentrations of EndoIII and EndoIII^Y82A^ (50 nM and 250 nM; Figure S6) were performed as described above. Also note that the same electrochemically oxidized (600 s, *E*_appl_ = 412 mV vs NHE) Pol2_CORE_exo^–^ sample was used for assays Ox, Ox+EndoIII, and Ox+EndoIII^Y82A^ in both concentrations.

### DNA Substrates for Primer Extension Assay

20-mer with 5′ tetrachlorofluorescein (TET) modification: 5′ TET-CGA GCC GTC TAC TCA ACT CA 3′.

40-mer with 5′ tetrachlorofluorescein (TET) modification: 5′ TET- CGA GCC GTC TAC TCA ACT CAT CCA GAA CAA CGT CAC TGA C 3′.

50-mer (well matched): 5′ CAG CTT GAT AGT CAG TGA CGT TGT TCT GGA TGA GTT GAG TAG ACG GCT CG 3′.

## Supporting Information Available

CV data of Pol2_CORE_exo^–^, Pol2_CORE_exo^–^CysX_MUT_, and DNA buffer; bulk oxidation yield curve; SDS–PAGE primer extension assay image (20:50 primer:template); and primer extension assay comparing activity of Pol2_CORE_exo^–^ samples treated with EndoIII (50–250 nM) and EndoIII^Y82A^ (50–250 nM) results (PDF)
