# Supplementary material for: The [4Fe4S] Cluster of Yeast DNA Polymerase ε Is Redox Active and Can Undergo DNA-Mediated Signaling
Source: J Am Chem Soc. 2021 Sep 24;143(39):16147–53. doi: 10.1021/jacs.1c07150 (PMC8499023; doi:10.1021/jacs.1c07150)
Supplement: Supplementary file 1 — ja1c07150_si_001.pdf [file ja1c07150_si_001.pdf]

Supporting Information for

The [4Fe4S] Cluster of Yeast DNA Polymerase ε is Redox Active and can undergo DNA-mediated Signaling

Miguel N. Pinto,[1] Josy ter Beek,[2] Levi A. Ekanger,[1,3]
Erik Johansson,[2]* and Jacqueline K. Barton[1]*

[1]Division of Chemistry and Chemical Engineering,
California Institute of Technology, Pasadena, CA 91125, USA
[2]Department of Medical Biochemistry and Biophysics,
Umeå University, SE-901 87, Umeå, Sweden
[3]Department of Chemistry, The College of New Jersey,
Ewing, NJ 08628, USA

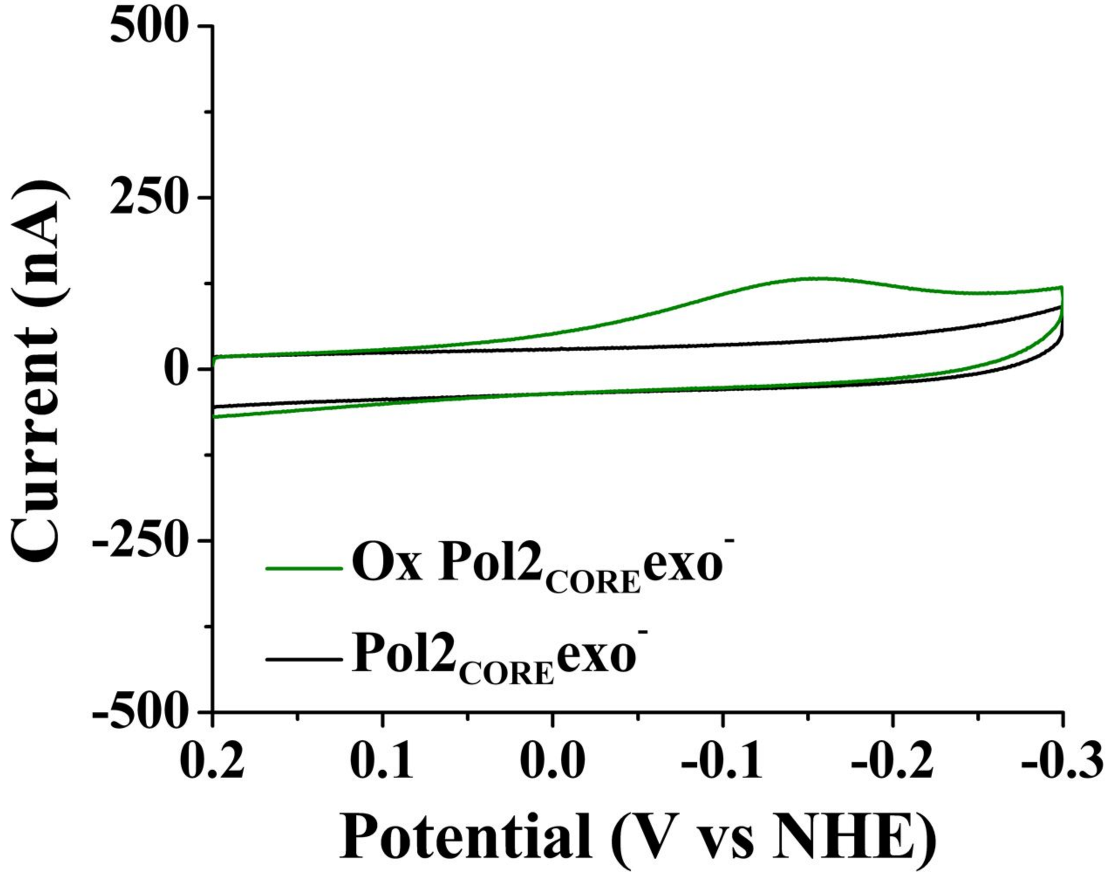

Figure S1. CV scans of electrochemically oxidized Pol2$_{CORE}$exo$^-$ (5 μM with respect to [4Fe4S], in 5 mM NaH_2PO_4, 50 mM NaCl, pH 7.0) exhibit a large cathodic CV signal centered around -140 mV vs NHE (green trace), and no signal for the electrochemically unaltered protein (black trace). Potential applied (E_{appl}) for bulk oxidation = 412 mV vs NHE for 500 s; CV scan rate = 100 mV s^{-1}.

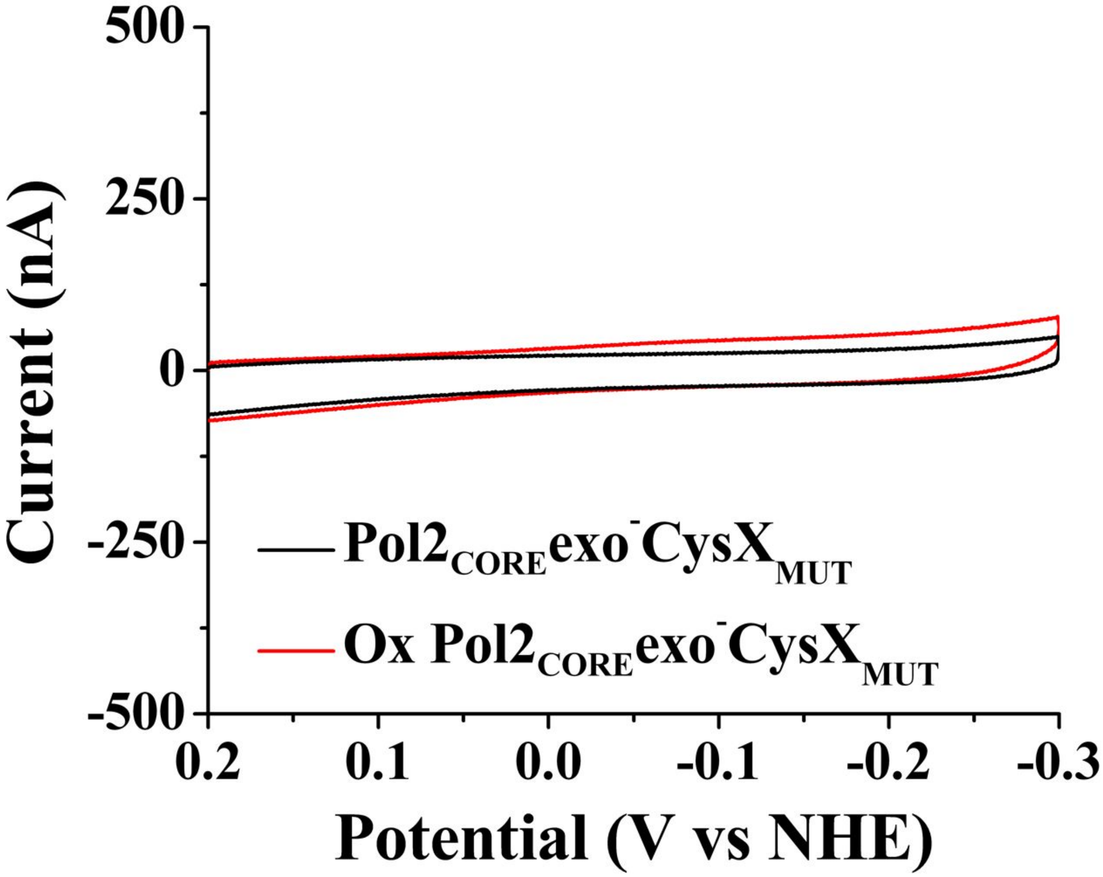

Figure S2. CV scans of electrochemically oxidized $Pol2_{CORE}exo^{-}$ $CysX_{MUT}$ (in 5 mM NaH_2PO_4, 50 mM NaCl, pH 7.0) does not exhibit a significant cathodic or anodic CV signal (red trace) compared to electrochemically unaltered protein (black trace). Potential applied (E_{appl}) for bulk oxidation = 412 mV vs NHE for 500 s; CV scan rate = 100 mV s^{-1}.

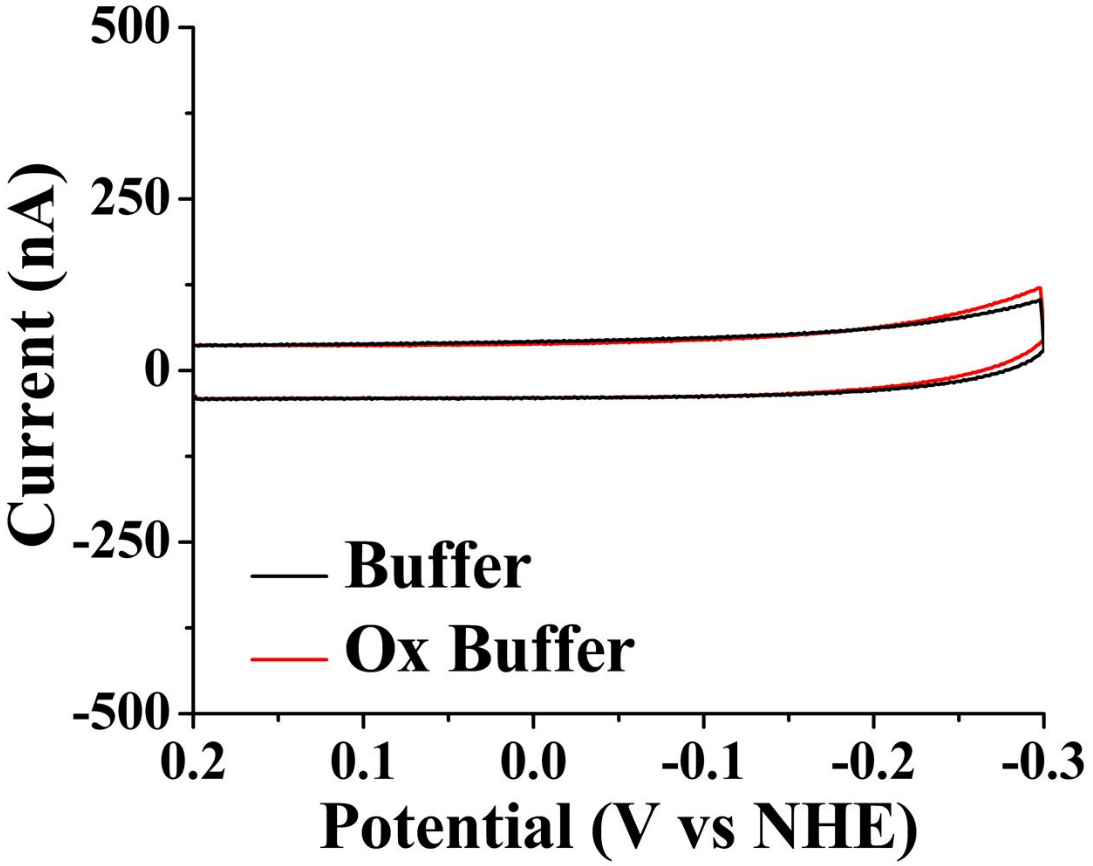

Figure S3. CV scans of electrochemically oxidized buffer (5 mM NaH_2PO_4, 50 mM NaCl, pH 7.0) does not exhibit a significant cathodic or anodic CV signal (red trace) compared to electrochemically unaltered protein (black trace). Potential applied (E_{appl}) for bulk oxidation = 412 mV vs NHE for 500 s; CV scan rate = 100 mV s^{-1}.

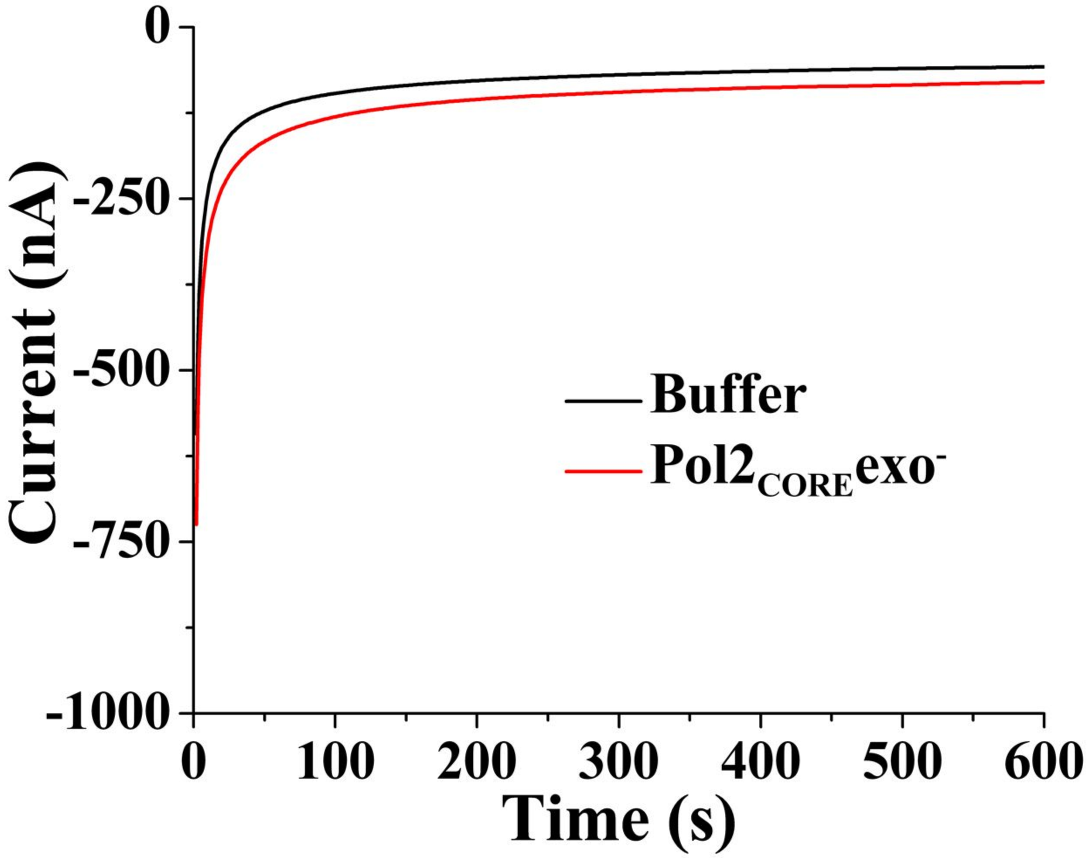

Figure S4. Characterization of electrochemically oxidized $Pol2_{CORE}exo^-$. Electrolysis of 25 μL of 5.0 μM $Pol2_{CORE}exo^-$ at 412 mV for 600 s gave ~95% oxidation yield. Bulk oxidation yields were calculated[1] by subtracting the amount of charge (reported in Coulombs) obtained from electrolysis of the buffer alone (black trace) from amount of charge obtained from electrolysis of the sample containing $Pol2_{CORE}exo^-$ (red trace). The resulting integrated area was converted to moles of electrons transferred using Faradays constant (96,485 C mol^{-1}). The obtained moles of electrons transferred equals the moles of oxidized $Pol2_{CORE}exo^-$ because oxidation of the [4Fe4S] cluster is a one-electron redox process. Finally, the bulk oxidation yield was obtained as a percentage by dividing the moles of oxidized [4Fe4S] cluster by the total moles of $Pol2_{CORE}exo^-$ contained in the 25 μL sample. Note: the total moles of $Pol2_{CORE}exo^-$ were obtained using its [4Fe4S] cluster absorbance (ε = 17,000 M^{-1} cm^{-1}).

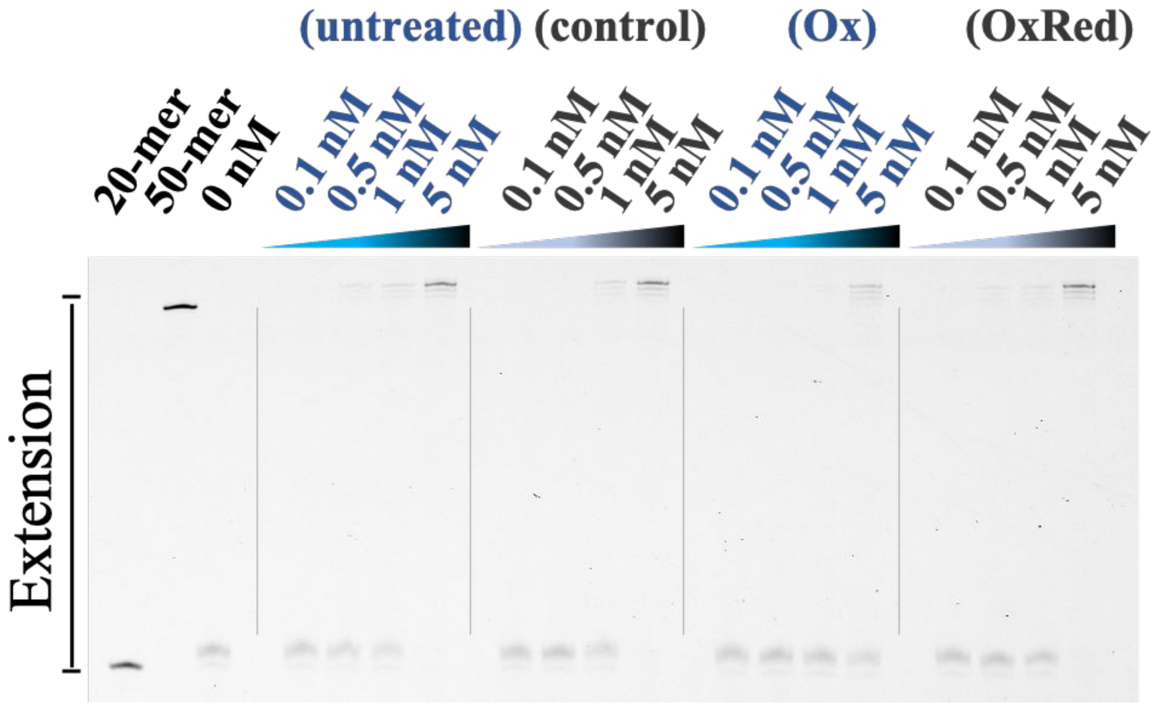

Figure S5. Representative example of denaturing PAGE results of Pol2$_{CORE}$exo$^-$ primer extension assay upon [4Fe4S] cluster oxidation/reduction. (untreated) activity of untreated Pol2$_{CORE}$exo$^-$; (control) activity of the wild type Pol2$_{CORE}$exo$^-$ after incubation on DNA modified electrodes without applied potential; (Ox) activity of Pol2$_{CORE}$exo$^-$ after bulk oxidation (600 s, E_{appl} = 412 mV vs NHE); (OxRed) activity of Pol2$_{CORE}$exo$^-$ after bulk oxidation (600 s, E_{appl} = 412 mV vs NHE) followed by bulk reduction (600 s, E_{appl} = -250 mV vs NHE). Bulk oxidation and bulk reduction were performed on 20 µL of 20 nM Pol2$_{CORE}$exo$^-$ using DNA-modified electrodes; all experiments were carried out in triplicate.

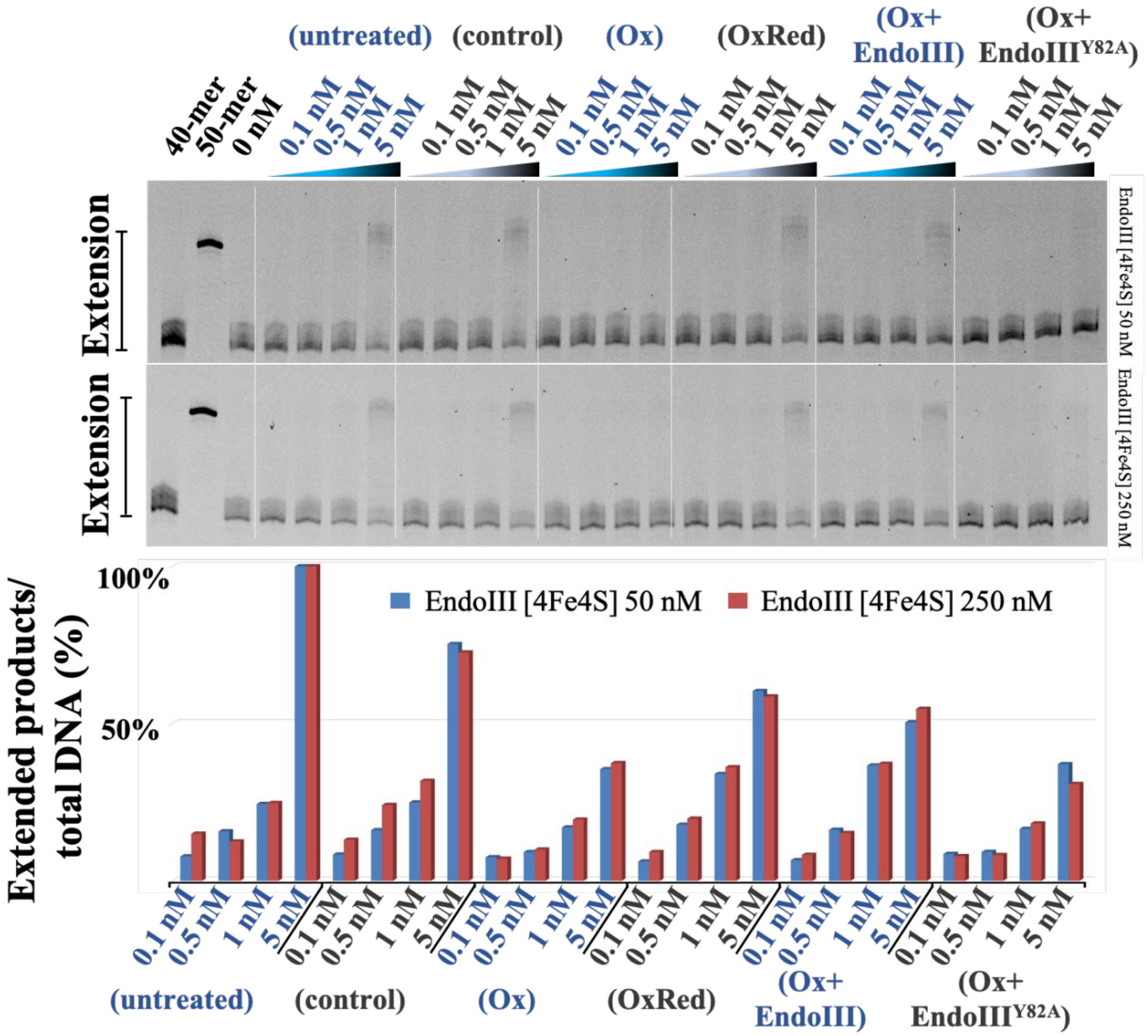

Figure S6. Primer extension assay investigating the effect of EndoIII and EndoIII^{Y82A} concentration on electrochemically oxidized and inactive Ox Pol2$_{CORE}$exo$^-$ using DNA-modified electrodes. (top panel) denaturing PAGE with products from Pol2$_{CORE}$exo$^-$ primer extension assays using 10x (upper gel image) and 50x (lower gel image) molar excess of reduced EndoIII (with respect to [4Fe4S]) (bottom panel), respectively. Bar graph presenting the quantified products in the denaturing PAGE (top two panels). (untreated) activity of untreated Pol2$_{CORE}$exo$^-$; (control) activity of Pol2$_{CORE}$exo$^-$ after incubation on DNA modified electrodes without an applied potential; (Ox) activity of Pol2$_{CORE}$exo$^-$ after bulk oxidation (600 s, E_{appl} = 412 mV vs NHE); (OxRed) activity of Pol2$_{CORE}$exo$^-$ after bulk oxidation (600 s, E_{appl} = 412 mV vs NHE) followed by bulk re-reduction (600 s, E_{appl} = -250 mV vs NHE). (Ox+EndoIII) is the activity of oxidized Pol2$_{CORE}$exo$^-$ after incubation with 10x (50 nM) or 50x (250 nM) EndoIII. (Ox+EndoIII^{Y82A}) is the activity of oxidized Pol2$_{CORE}$exo$^-$ after incubation with 10x (50 nM) or 50x (250 nM) EndoIII^{Y82A}. It should be noted that experiments with Ox, Ox+EndoIII, and Ox+ EndoIII^{Y82A} in both concentrations were performed the same day/time using the same oxidized Pol2$_{CORE}$exo$^-$ sample. Bulk oxidation and bulk reduction were performed on 20 μL of 20 nM Pol2$_{CORE}$exo$^-$ using DNA-modified electrodes. all experiments were carried out in triplicate, error bars indicate standard deviation.

REFERENCES

(1) (a) Bartels, P. L.; Stodola, J. L.; Burgers, P. M. J.; Barton, J. K. A Redox Role for the [4Fe4S] Cluster of Yeast DNA Polymerase δ. *J. Am. Chem. Soc*. **2017**, *139*, 18339 – 18348. (b) O'Brien, E.; Holt, M. E.; Thompson, M. K.; Salay, L. E.; Ehlinger, A. C.; Chazin, W. J.; Barton, J. K. The [4Fe4S] Cluster of Human DNA Primase Functions as a Redox Switch Using DNA Charge Transport. *Science* **2017**, *355*, 813. (c) O'Brien, E.; Salay, L. E.; Epum, E. A.; Friedman, K. L.; Chazin, W. J.; Barton, J. K. Yeast Require Redox Switching in DNA Primase. *Proc. Natl. Acad. Sci*. **2018**, *115*, 13186 – 13191. (d) Tse, E. C. M.; Zwang, T. J.; Barton, J. K. The Oxidation State of [4Fe4S] Clusters Modulates the DNA-Binding Affinity of DNA Repair Proteins. *J. Am. Chem. Soc*. **2017**, *139*, 12784 – 12792. (e) Grodick, M. A.; Segal, H. M.; Zwang, T. J.; Barton, J. K. DNA-Mediated Signaling by Proteins with 4Fe-4S Clusters is Necessary for Genomic Integrity. *J. Am. Chem. Soc*. **2014**, *136*, 6470 – 6478.
